# Recurrent hypermotif regulatory circuits in developmental programs

**DOI:** 10.1073/pnas.2538056123

**Published:** 2026-10-05

**Authors:** Miri Adler, Ruslan Medzhitov

**Affiliations:** ^a^ Department of Genetics, Silberman Institute of Life Science, The Hebrew University of Jerusalem, Jerusalem 9190401, Israel; ^b^ Department of Immunology and Cancer Research, Faculty of Medicine, The Hebrew University of Jerusalem, Jerusalem 9112102, Israel; ^c^ Tananbaum Center for Theoretical and Analytical Human Biology, Yale University School of Medicine, New Haven, CT 06510; ^d^ Department of Immunobiology, Yale University School of Medicine, New Haven, CT 06510; ^e^ HHMI, Yale University School of Medicine, New Haven, CT 06510

**Keywords:** gene regulatory networks, network motifs, morphogenes

## Abstract

During development, cells self-organize into complex spatial and temporal patterns driven by regulatory circuits of transcription factors and morphogen signaling. However, the principles that govern these regulatory interactions remain poorly understood. Here, we introduce a framework to dissect the building-block circuits of developmental gene regulatory networks and explore their integration into higher-order hypermotif circuits. Using single-cell RNA-sequencing data spanning human intestinal development, we identify five recurrent network motifs, including feedforward and feedback loops, and investigate their roles in the regulation of gene expression. Our analysis reveals distinct categories of developmental genes based on their roles within network motifs, highlighting major transitions in regulatory architecture across developmental stages. Furthermore, we model the emergent dynamical properties of hypermotif circuits, including their potential roles in regulating morphogen signaling. This study uncovers a common theme in regulatory circuits of developmental programs, emphasizing how the combinatorial wiring of regulatory motifs enables robust and diverse tissue formation.

## Significance

Embryonic development is a tightly orchestrated process in which cells decide when and where to differentiate, divide, migrate, or die to build healthy tissues. Remarkably, a relatively small set of genes is reused across stages and across organs, raising a fundamental question: how can the same limited toolkit generate diverse tissue patterns? These genes form dense regulatory networks, but it is difficult to interpret which interactions are essential and what design rules ensure robust outcomes. Here we identify recurring small regulatory circuits (motifs) and map how they combine into higher-order hypermotifs, analogous to a minimal set of LEGO pieces assembly rules needed to create diverse forms. This framework highlights core genes and links likely critical for normal development and disease.

**Fig. 1.**
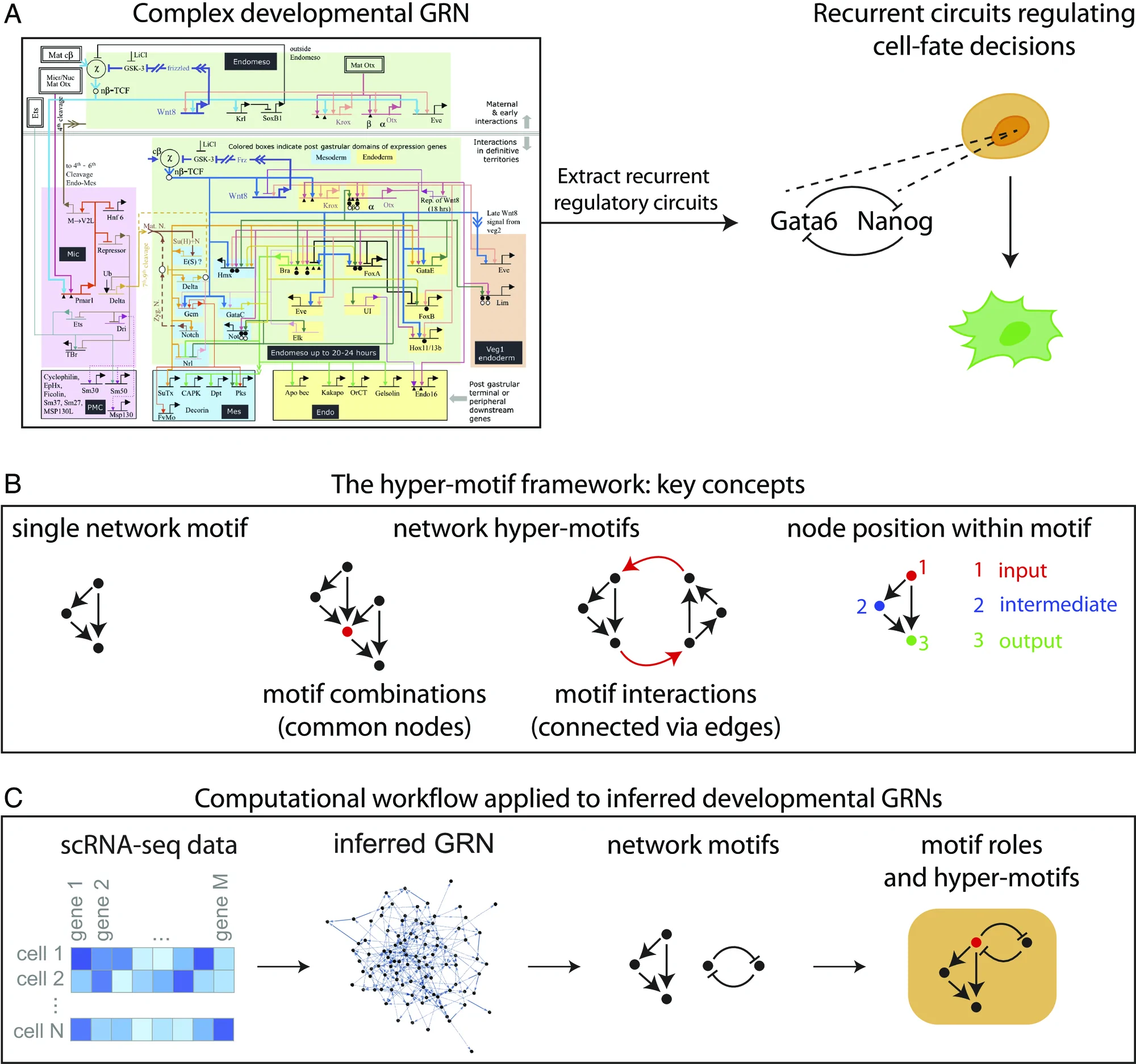
A network analysis framework to explore developmental programs. (*A*) Out of the multitude of regulatory interactions in developmental GRNs, what are the building-block circuits of developmental programs that are important for cell-fate decisions? Complex developmental GRNs can be decomposed into recurrent regulatory circuits. [GRN adapted from Davidson et al. (24)]. (*B*) Definition of the hypermotif framework: given two network motifs, their hypermotifs are defined as combinations of motifs where the motifs share at least one node or interactions of motifs where the motifs are directly linked through at least one edge. Distinguishing different node positions within the motif is important to explore different hypermotif structures. (*C*) A computational framework to analyze regulatory networks by identifying network motifs as building-block circuits and how they are joined together to form higher-order network hypermotifs within cells. Starting from scRNA-seq data, we infer developmental GRNs, identify recurrent network motifs, and analyze motif roles (node positions within motifs) and hypermotif structures. Image credit: Adapted from ref. 24.

Embryonic development is a well-orchestrated process in which cells divide, migrate, differentiate, and self-organize into the different tissues that form the embryo. Starting from a single fertilized egg, cells use communication signals that they secrete and sense to regulate their cell fate decisions (1). These signals, also known as morphogens, form gradients that inform the cells of their position and allow them to respond accordingly (2–6). Several families of morphogens play key roles in shaping the spatial patterns in the embryo, including the bone morphogenic proteins (BMPs) (7, 8), fibroblast growth factors (FGFs) (9), Hedgehog (HH) (10, 11), WNT (12, 13), and Retinoic acid (14). The expression of these morphogens and their receptors is regulated by several transcription factor (TF) families that play critical roles in development, including HOX, GATA, ETS, SOX, PAX, TBOX, FOX, and E-proteins (15–23). These morphogens and TFs regulate each other’s expression through intracellular regulatory circuits as well as cell-cell communication circuits, forming a complex network of interacting genes (24–31) (Fig. 1*A*). While much is known about individual signaling pathways and transcriptional regulators, the overarching principles governing these interactions remain unclear. Two complementary conceptual frameworks provide guiding principles governing cell fate decisions: the Turing reaction-diffusion model and the concept of positional information (32–37). Both models highlight the role of regulatory circuits in interpreting spatial and temporal cues to drive developmental patterning.

Studies in model organisms have provided insights into key pathways in developmental processes, where a detailed transcription network can be inferred in specific contexts from experimental data (as exemplified in Fig. 1*A*). However, it remains unclear which circuits out of the detailed transcription network are the “building blocks” required for the proper development of the organism, and how their dynamical properties explain the wide range of observed tissue-level patterns (38).

Previous theoretical and experimental work has defined network motifs, which are recurrent patterns of regulatory interactions, as building blocks of gene regulatory networks (GRNs) (39, 40). Network motifs such as feedforward loops (FFLs) have been extensively studied, and their dynamical properties were elucidated (41, 42). Seminal previous work has uncovered several families of network motif designs that can provide distinct behaviors in the interpretation of morphogen gradients (43). However, how these motifs integrate within the broader regulatory network to influence their function remains poorly understood. The combination of small network motifs into larger structures may generate emergent properties that cannot be explained by individual motifs alone. Therefore, a comprehensive understanding of developmental GRNs requires an investigation of how motifs interact and organize into higher-order structures.

To address this, we recently introduced the concept of hypermotifs, higher-level network modules that describe how individual motifs are embedded within larger networks (44). Hypermotifs are formed when multiple network motifs share at least one common node (combinations of motifs) or are directly linked through regulatory interactions (motif interactions) (Fig. 1*B*). These structures can generate novel emergent properties not present in individual motifs (45). Such properties are likely involved in controlling developmental timing, cell fate decisions, and spatial patterning during embryogenesis.

Here, we develop a network analysis framework to uncover the principles governing gene regulatory interactions in developmental programs. While we focus primarily on the transcriptional level in this analysis, we acknowledge the critical roles of other regulatory mechanisms, including posttranslational modifications and protein-protein interactions. Nevertheless, a substantial understanding can still be gained by focusing on transcription regulatory networks. This approach is analogous to a mathematical “projection” of a high-dimensional object, encompassing all types of causal interactions, into a lower-dimensional subspace that reflects transcription factor-DNA binding and its effects on transcription. Although this projection does not capture some features, it retains sufficient causal structure to provide significant insights into the system.

We explore how the different network motifs in developmental programs are wired together forming higher-level network patterns (Fig. 1*C*). These higher-level structures can form hypermotif regulatory circuits within cells, which are the main focus of this study, or hypermotif circuits between different cells that are connected through morphogen signaling pathways, which is the main focus of the accompanying study in Adler et al. This framework can help explain how regulatory interactions that are governed by a minimal set of transcription-factor regulators show diverse, complex, and precise emergent properties.

## Results

### Enriched Network Motifs in the Development of the Human Intestine.

**Fig. 2.**
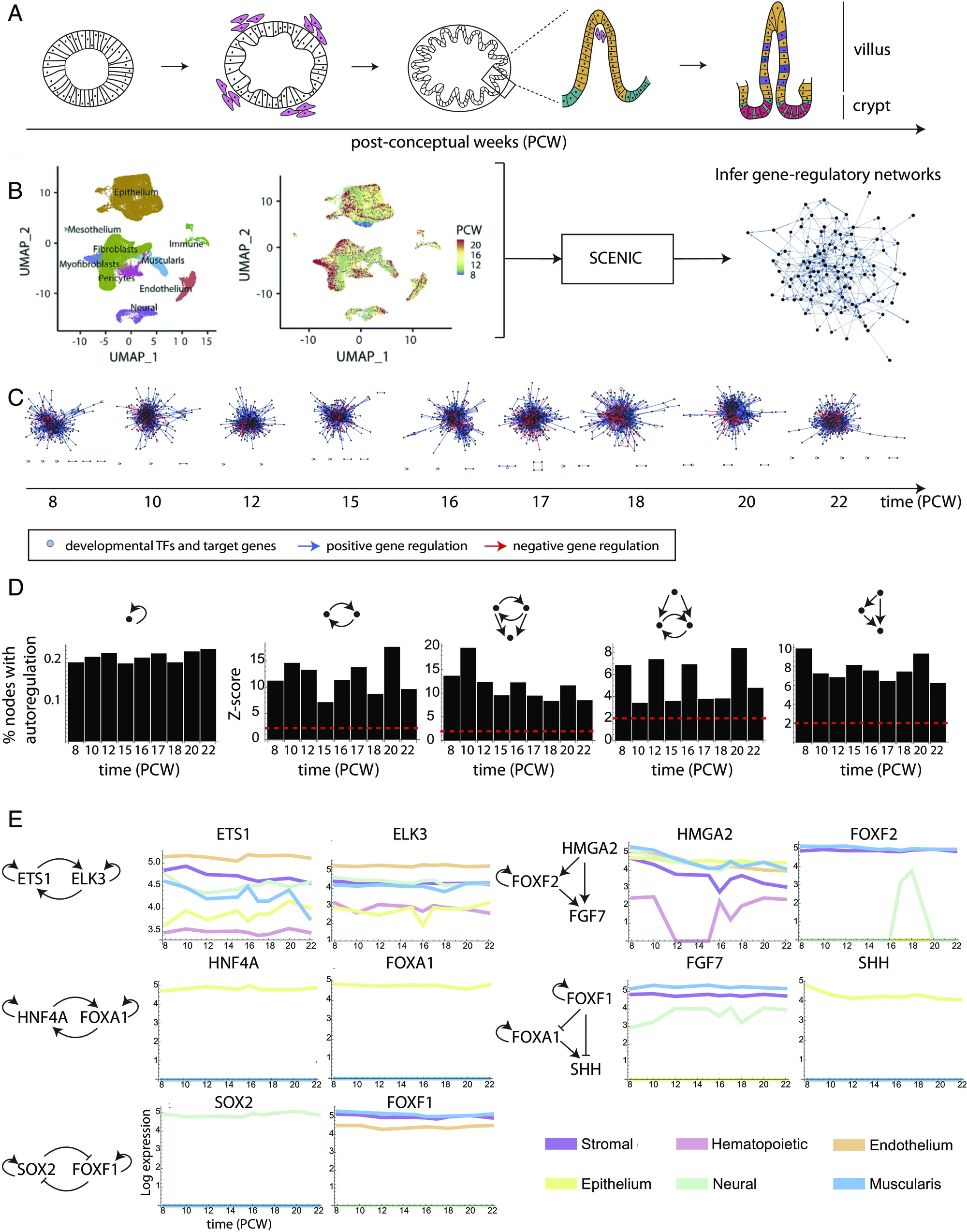
Five network motifs enriched in GRNs of developing intestine. (*A*) Schematic of intestine development that is spanned by the data in Fawkner-Corbett et al. (46). (*B*) We analyze the single-cell data and use SCENIC to infer gene regulatory interactions between developmental-related TFs and target genes. (*C*) The GRNs inferred from the data across 9 developmental time points. Nodes are TFs and target genes, edges represent positive (blue) and negative (red) interactions. (*D*) The most statistically enriched network motifs are shown with their Z-score. The red dashed line is a threshold for enrichment significance. Because autoregulation corresponds to self-loops, which are constrained by the degree-preserving randomization procedure, autoregulation is quantified separately as the fraction of nodes with self-regulation rather than by a Z-score. (*E*) Examples of network motifs inferred from 8 PCW. Log mean expression of genes in 6 major cell type categories is plotted as a function of time (PCW).

We analyzed single-cell RNA-sequencing (scRNAseq) data from the developing human intestine across nine time points, spanning 8 to 22 postconception weeks (PCWs), as reported by Fawkner-Corbett et al. (46) (Fig. 2 *A* and *B*). Our analysis focused on developmental genes, including transcription factors (TFs), morphogen ligands, their antagonists, receptors, and coreceptors. To infer regulatory interactions between developmental TFs and their target genes, we employed SCENIC (47) (Fig. 2*C* and *Methods*). The overall size of the GRNs remained relatively stable across developmental time points, with the number of genes ranging between 220 and 275 and the number of edges fluctuating between 752 and 1,045 (*SI Appendix*, Fig. S1).

Across all time points, we identified five network motifs (of up to 3-node circuits, *Methods*) that were consistently enriched. These motifs include: 1) autoregulation (predominantly positive), 2) mutual feedback loops, 3) *regulated* feedback loops, 4) *regulating* feedback loops, and 5) FFLs (Fig. 2*D*). These motifs have been observed in developmental programs across multiple organisms (42), supporting the biological relevance of the enriched motif repertoire. The dynamic properties of these motifs, such as bistability and delayed response time, are crucial for proper developmental progression (48, 49). To further examine whether motif enrichment reflects selective network organization rather than a general enrichment of all small topologies, we also analyzed underrepresented motifs, or anti-motifs. Four three-node topologies show consistently negative Z-scores relative to degree-preserving randomized networks across developmental time points (*SI Appendix*, Fig. S2), indicating that developmental GRNs display both recurrent enrichment and reproducible depletion of specific circuit architectures.

Genes that participate in these inferred motifs can show diverse expression patterns, ranging from commonly expressed genes to cell type-specific expression (Fig. 2*E*). Our inferred networks aggregate all regulatory interactions from genes expressed across multiple cell types, capturing global regulatory trends. Notably, we observed similar motif enrichment patterns when analyzing regulatory interactions within individual cell types (*SI Appendix*, Fig. S3), suggesting that these motifs are fundamental components of developmental regulatory programs.

### Developmental Genes Can Be Categorized Based on Their Position in the Network Motifs.

**Fig. 3.**
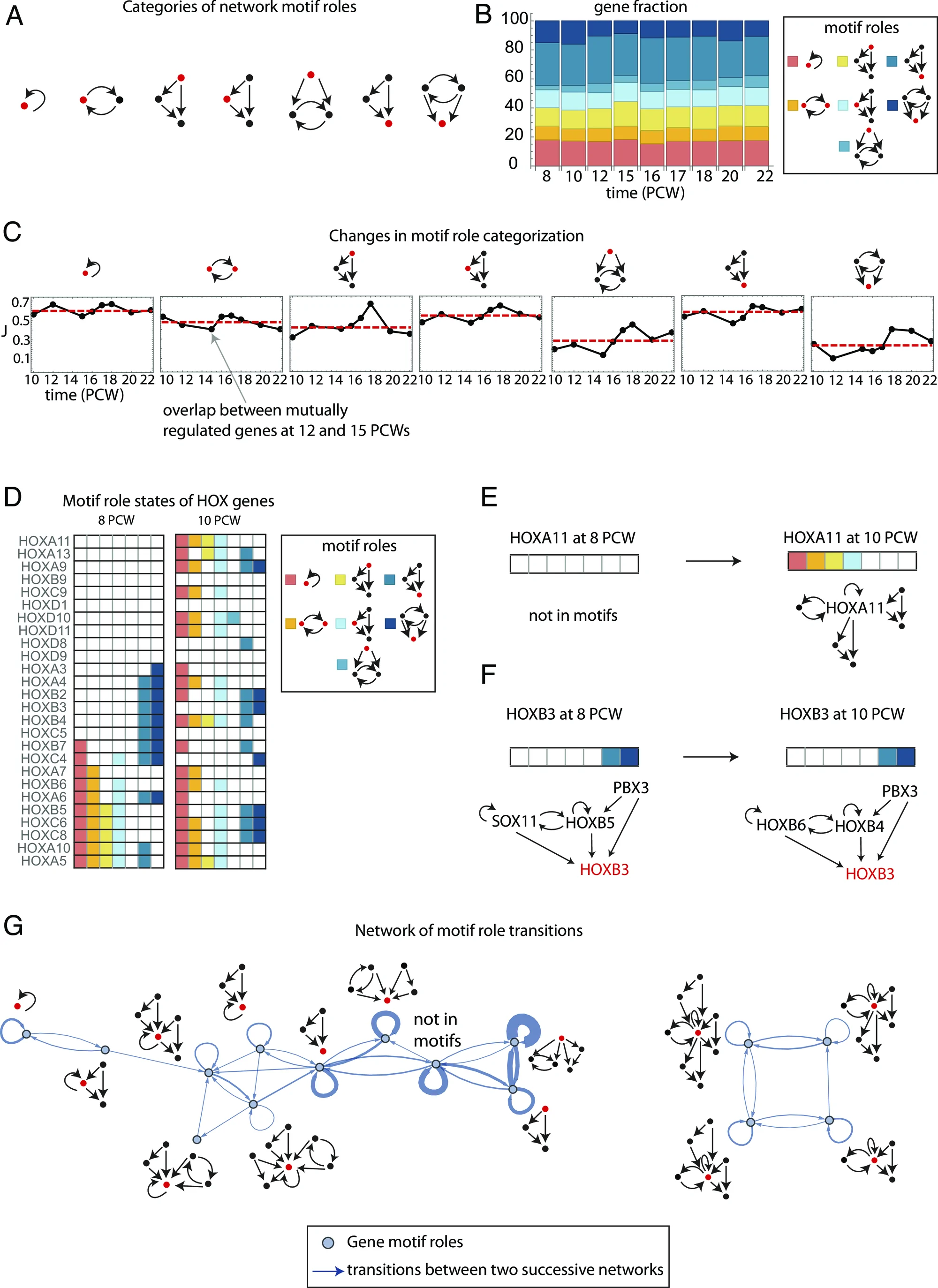
Categorizing developmental genes by motif roles highlights developmental transitions. (*A*) Developmental genes can be categorized to 7 motif roles (highlighted in red). (*B*) Fraction of genes participating in each motif role at each time point. (*C*) In each panel the black points are the Jaccard index between the two sets of genes participating in a particular motif role from two successive time points. The red dashed line is the median Jaccard index across all time points. (*D*) Table of motif role states of HOX genes at 8 and 10 PCWs. (*E*) HOXA11 transitions from not participating in motifs to a node common to multiple network motifs combined. (*F*) HOXB3 maintains the same motif role state between 8 and 10 PCWs despite other genes in the motif that change their regulatory roles. (*G*) Network of motif role transitions. Nodes in the network represent motif role states and edges represent transitions between two successive networks. Only transitions that occur more than 2 times are considered.

To investigate patterns in network structure over time, we categorize genes at each time point based on their position within network motifs (referred to as their motif role, Fig. 1*B*). This approach allows us to explore whether genes retain their motif roles over time or transition between different positions within the network. In the context of intestinal developmental programs, we identify five network motifs that collectively define seven distinct motif roles: autoregulated TFs; TFs in mutual feedback; input, intermediate, and output positions within an FFL; input to a regulated feedback loop; and output of a regulating feedback loop (Fig. 3*A*). By categorizing genes into these seven motif roles, we observe that the most prevalent role across networks is the output position of an FFL (Fig. 3*B*). Additionally, analyzing the proportions of genes in input, intermediate, and output roles reveals that intermediate genes form a significantly smaller group than input and output genes, suggesting a bowtie structure in the networks (50).

To explore whether motif-role assignment provides a temporally stable classification of developmental genes, we examine changes in the TFs and genes occupying each role over time. Using the Jaccard index (*Methods*), we measure the overlap between gene lists assigned to a specific motif role in successive time points (Fig. 3*C*). We find that autoregulation is the most stable motif role, with approximately 60% overlap between autoregulated TFs in consecutive time points, whereas roles such as input to a regulated feedback loop and output of a regulating feedback loop show greater turnover, with only ~30% overlap between consecutive time points (Fig. 3*C*). These values reflect preservation between adjacent developmental stages, rather than maintenance of the same genes throughout the full developmental process. Extending the analysis to nonconsecutive time points shows that gene conservation within motif roles decreases with increasing developmental distance. However, autoregulation remains the most stable role, with 22% of autoregulated TFs maintaining this role throughout the time course. In contrast, only 2% of genes remain input nodes to a regulated feedback loop throughout development. This analysis further shows that motif-role identities shift more strongly at earlier developmental stages and become more stable after 16 PCW (*SI Appendix*, Fig. S4*A*).

This analysis highlights time points where larger compositional changes occur in the genes occupying a given motif role. Intervals with Jaccard values below the median show relatively higher turnover in motif-role identity (red dashed line in Fig. 3*C*). Notably, the largest compositional shifts in the regulated feedback input occur at 10, 12, and 15 PCWs (Fig. 3*C*, second panel from the *Right*).

To understand the biological significance of these transitions, we analyze the TFs and genes that are added to specific motif roles at these critical time points. At 10 PCW, genes added include *BMP2, BMP4,* and *BMP5*, which are known to be expressed in precryptal S2 fibroblasts (telocytes) and contribute to epithelial proliferation and differentiation (46). Other additions at this stage include *FOXD1*, which regulates terminal ileum development, *TCF21*, important for early fibroblast development, and *TWIST2*, a key regulator of muscularis cell differentiation. By 12 PCW, *FOXF2*, which plays a crucial role in muscle cell differentiation, appears in a new motif role. At 15 PCW, several epithelial-restricted genes emerge, including *LEFTY1* and *IHH*, which are part of an epithelial-specific module. At 16 PCW we find WNT5B, which is expressed by stromal, neuronal, and muscle cells and supports epithelial cells. At 20 PCW, *FOXL1*, a marker for S2 fibroblast differentiation, and *WNT2B*, expressed in a myofibroblast module, emerge. Finally, at 22 PCW, *SOX13*, which plays a role in intestinal angiogenesis, is introduced in a new motif role (46).

To determine whether motif role transitions occur randomly or follow specific rules, we define a “motif role state” for each gene, capturing its participation in one or multiple motif roles simultaneously (forming a hypermotif as discussed next). By tracking motif role states over time, we identify distinct patterns of transition. For example, in the case of *HOX* genes within our inferred networks (Fig. 3*D*), some maintain the same motif role state across time (Fig. 3*F*), while others transition into new roles (Fig. 3*E* and *SI Appendix*, Fig. S2).

To systematically examine these transitions, we construct a network of motif role state transitions (Fig. 3*G*). In this network, nodes represent motif role states, defined as binary combinations of the seven motif roles, and edges denote transitions between states across successive time points, with edge weights reflecting transition frequencies. Consistent with the nonrandom organization of motif-role combinations, only 46 of the $2^{7}=128$ possible motif-role states are observed at least once, and the number of recurrent states remains on the order of a few tens across a range of frequency thresholds (*SI Appendix*, Fig. S4*B*). To focus on recurrent transitions, Fig. 3*G* includes only transitions observed more than twice. The full unfiltered transition network contains many additional low-frequency edges (*SI Appendix*, Fig. S4*C*). Thus, missing edges in the filtered network, including the separation into two components, should not be interpreted as evidence that such transitions cannot occur, but rather as transitions that are relatively rare. The recurrent transition network suggests that motif-role changes are not uniformly distributed across state space, but instead follow preferred transition patterns. For example, input genes do not commonly transition directly into output genes and vice versa, and TFs involved in mutual feedback loops often serve as network hubs, acting as common nodes across multiple motifs (Fig. 3*G*).

### Network Motif Identity Captures Cell-State Similarity.

**Fig. 4.**
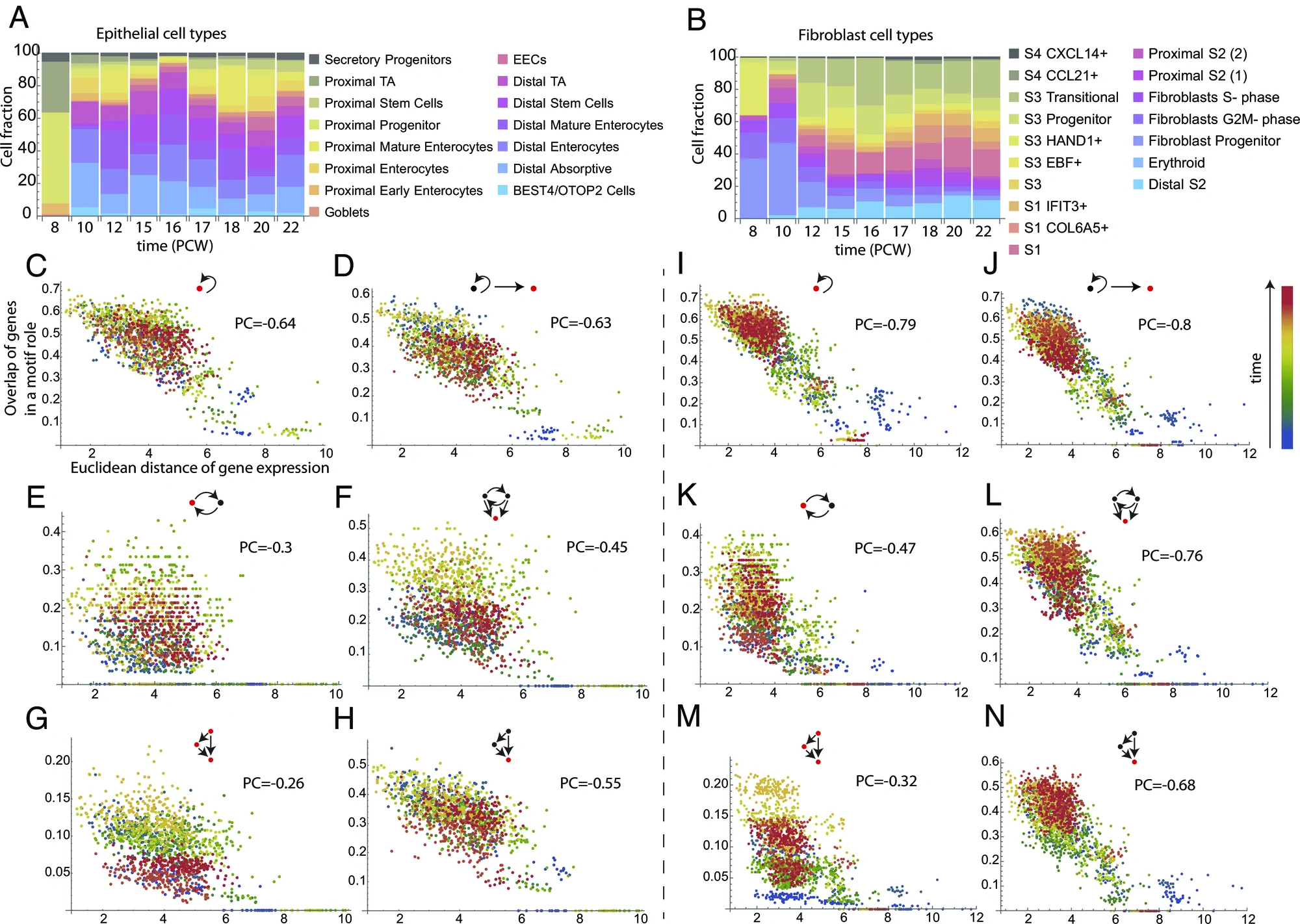
High correlations between network motif identity and gene expression distance in epithelial and fibroblast cell types. (*A* and *B*) Cell fraction of epithelial (*A*) and fibroblast (*B*) cell types across time. (*C*–*N*) Every point is a pair of epithelial (*C*–*H*) or fibroblast (*I*–*N*) cell types, where the *y* axis is the overlap (Jaccard index) between genes participating in a network motif or its output gene in the two cell types and the *x* axis is the Euclidean distance between the gene expression profiles of the two cell types. The overlap in genes is computed for the nodes marked in red in each panel. The color of the points in the plots represents the developmental stage from early stage (8 PCW—blue) to late stage (22 PCW—red). PC = Pearson correlation.

To further examine the regulatory role of network motifs in developmental programs, we analyze the relationship between motif identity and similarity in gene expression profiles. We focus on epithelial and fibroblast cell populations, considering the cell types into which epithelial and fibroblast progenitors diversify (Fig. 4 *A* and *B*). Within each category, we calculate the Euclidean distance between every pair of cell types (e.g., secretory progenitors and goblet cells, proximal enterocytes, and proximal stem cells) in gene expression space based on their average transcriptome-wide gene expression profiles (*Methods*). We then compare this gene expression distance to the similarity in inferred network motifs regulating these cells’ gene expression.

To estimate network motif similarity, we compute the overlap of gene lists participating in or regulated by each motif between cell pairs (*Methods*). Our findings reveal a strong correlation between gene expression distance and network motif overlap: cell types that share more network motifs tend to have more similar gene expression profiles (Fig. 4 *C*–*N*). This association is particularly striking given that the overlap of a relatively small group of autoregulated TFs is strongly associated with transcriptome-wide gene expression similarity across ~20,000 genes (Fig. 4 *C* and *I*).

Notably, some network motifs show stronger associations with gene expression similarity than others. For instance, in epithelial cells, autoregulation exhibits a stronger correlation with gene expression than mutual feedback and FFLs (Fig. 4 *E* and *G*). Focusing on effector genes, those directly regulated by network motifs, further strengthens these correlations (Fig. 4 *D*, *F*, and *H*). Additionally, fibroblast cell types consistently show higher correlations across all examined network motifs and their effector genes compared to epithelial cells. Together, these results suggest that motif identity captures regulatory information associated with cell-state similarity, although perturbation experiments would be required to determine whether specific motifs or genes causally regulate these expression states.

### Recurrent Network Hypermotifs Reveal Common Patterns.

**Fig. 5.**
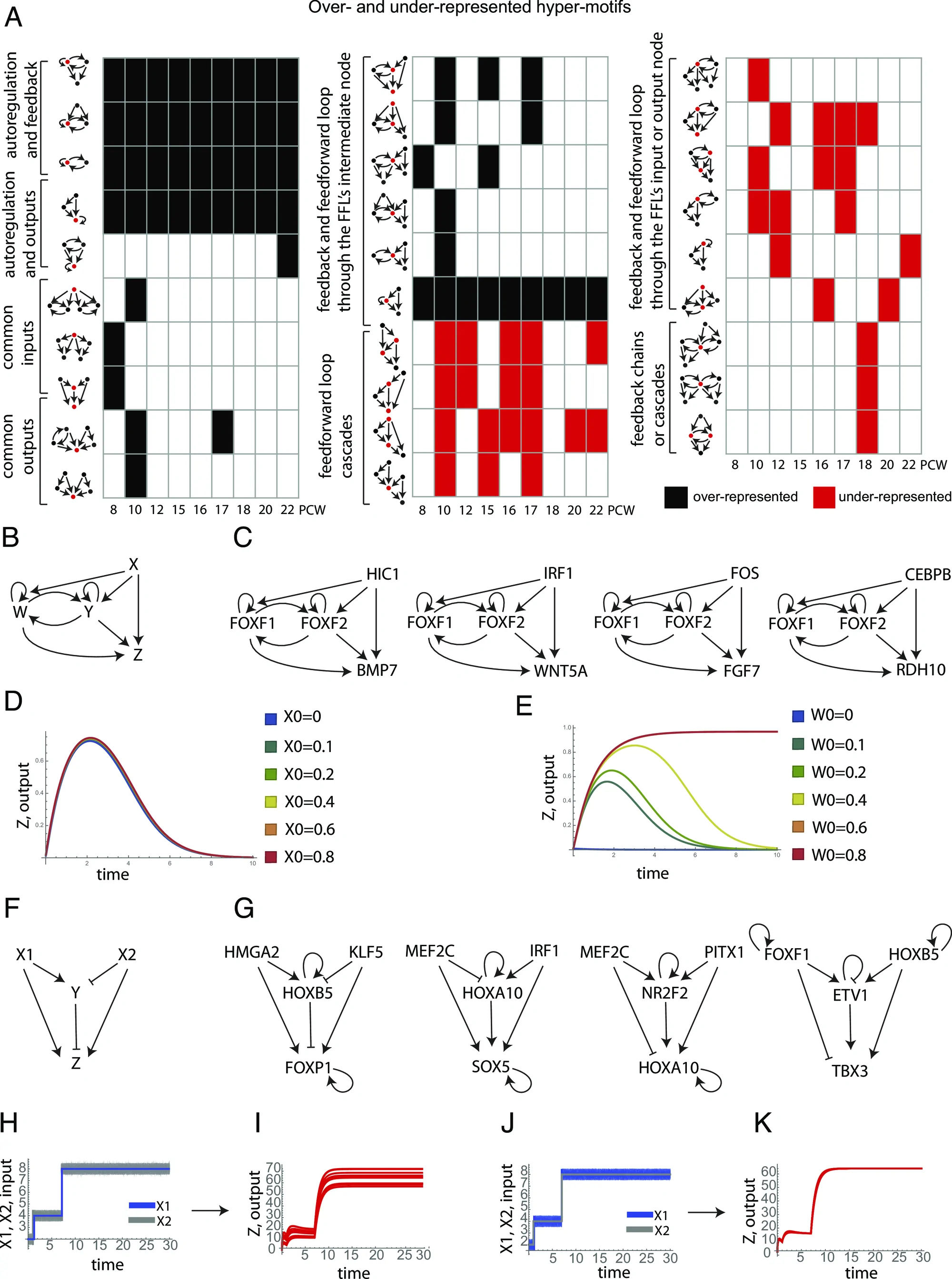
Enriched network hypermotifs and their dynamical behavior. (*A*) Table of over- and underrepresented hypermotifs across the developmental process. Rows are different hypermotif topologies; columns are different time points in the data. Black squares mark positive enrichment, and red squares mark negative enrichment. (*B*) An enriched hypermotif circuit where input signal X activates output gene Z and the mutually regulating TFs Y and W which both activate Z as well. (*C*) Examples of the hypermotif in the developmental networks. (*D* and *E*) Dynamical behavior of the output gene Z where the different curves are for different initial conditions of the input signal X (*D*) or the TF W (*E*). (*F*) Hypermotif circuit that combines a coherent FFL and an incoherent FFL. (*G*) Examples of the hypermotif in the developmental networks. (*H*) Simultaneous step-like changes in X1 (noisy, gray) and X2 (precise, blue) (*I*) Dynamical behavior of output Z in response to the input signals plotted in *H*. (*J-K*) Same as *H*–*I* only with X1 as the noisy signal and X2 as the precise signal.

Next, we identify the most statistically enriched hypermotifs (44), where multiple motifs are interconnected through at least one common node (*Methods*). These enriched hypermotifs exhibit recurring patterns: autoregulation frequently couples with feedback circuits or FFLs via their intermediate and output nodes, and feedback circuits often connect to FFLs through their intermediate nodes. Additionally, motifs frequently share a common input or output node (Fig. 5*A*).

Certain motif combinations are consistently enriched across all developmental time points, while others are significantly enriched only at specific stages. Because combinations of motifs can generate regulatory properties that differ from those of individual motifs, stage-specific enrichment of hypermotifs may reflect changes in the regulatory architecture of the developmental program. The largest number of enriched hypermotif topologies appears at 10 PCW, coinciding with the stage in which the crypt-to-villus axis is formed (Fig. 5*A*), suggesting an association between hypermotif organization and developmental remodeling.

Conversely, underrepresented network hypermotifs follow distinct patterns. Autoregulation and mutual feedback rarely combine with the input node of an FFL, multiple feedback circuits are selectively avoided at 18 PCW, and cascades of FFLs are absent at multiple time points (Fig. 5*A*). These findings highlight specific constraints shaping the regulatory network’s temporal evolution.

### Dynamical Properties of Enriched Hypermotif Regulatory Circuits.

We analyze several statistically enriched hypermotif regulatory circuits in the networks and develop mathematical models to investigate their dynamic behavior. These simulations are intended as proof-of-principle examples of dynamic behaviors that can be supported by the inferred hypermotif topologies, rather than an exhaustive characterization of all possible behaviors generated by these circuits. One prevalent hypermotif consists of a feedback circuit coupled with an FFL through the intermediate node of the FFL (Fig. 5*B*). This hypermotif is frequently observed in the regulatory networks of stromal and epithelial cells throughout development, directly influencing the expression of morphogen ligands, receptors, and various development-related TFs (Fig. 5*C*). We developed a mathematical model for the dynamical behavior of this hypermotif (*Methods*). Our model suggests an interesting property of this hypermotif where it integrates two distinct types of input signals that regulate the output gene. The first input is the FFL’s input *X*, while the second comprises the mutually regulating *Y/W* TFs. Although both inputs are necessary for *Z* transcription, they impact *Z*’s dynamics differently. Specifically, *X* is required to activate *Z*, but its initial or final levels do not significantly influence *Z*’s behavior, making *Z* insensitive to variations in *X* (Fig. 5*D*). In contrast, the initial levels of *Y* or *W* strongly affect *Z*’s dynamics (Fig. 5*E*). Thus, this hypermotif can differentially integrate two inputs: one input enables output activation, whereas the state of the feedback module determines the resulting output dynamics. This sensitivity enables distinct regulatory responses to varying input levels, aligning with mechanisms such as morphogen gradient interpretation.

Additionally, we examine a hypermotif that integrates coherent and incoherent FFLs (Fig. 5*F*). This circuit is observed, for example, at 8 PCWs in fibroblast cells, where the HMGA2-HOXB5-FOXP1 circuit functions as an incoherent FFL (HMGA2 directly activates FOXP1 while inhibiting it through HOXB5), whereas the KLF5-HOXB5-FOXP1 circuit forms a coherent FFL (Fig. 5*G*). This hypermotif retains known properties of individual FFLs when their input signals vary independently. However, when both input signals fluctuate simultaneously, the motif exhibits a distinctive behavior: the final output *Z* remains robust to variations in *X1*, which regulates the incoherent FFL, while being sensitive to noise in *X2*, the input for the coherent FFL (Fig. 5 *H*–*O*). In this parameter regime, the hypermotif therefore buffers variation in one input while preserving sensitivity to another input. This dual-mode regulation suggests that hypermotifs contribute to robustness and adaptability in GRNs. Other dynamic behaviors, including delayed responses or fold-change detection, may occur under different parameter regimes, and the examples shown here illustrate selected qualitative capabilities of these circuit topologies.

## Discussion

Developmental processes are regulated by complex regulatory networks. To disentangle this complexity, we used our recently developed framework that examines how fundamental building-block circuits integrate into larger networks, providing insight into the structure and function of developmental gene regulatory programs. Applying this method to single-cell intestinal development data, we identified five core network motifs that assemble according to distinct rules. We found that motif identity and gene position within motifs strongly predict gene expression profiles, particularly in epithelial and fibroblast populations, underscoring the utility of the network motif approach in deciphering GRNs. Moreover, key transitions in network structure, such as the emergence of enriched hypermotifs and the incorporation of novel genes into critical network positions, highlight major developmental events and their potential regulatory drivers.

This framework introduces a way to classify developmental genes based on their functional roles within network motifs, distinguishing input genes, effector genes, and hub genes that integrate multiple motifs to coordinate regulatory control. Extending this approach to other tissues and developmental processes could further validate the robustness and generalizability of network motif-based gene classification.

In our analysis of over- and underrepresented network hypermotifs, we found that positive feedback circuits, including autoregulatory and mutual feedback, were highly enriched throughout development, whereas feedforward loop cascades were underrepresented. Although both motifs contribute to delayed responses, an important property in developmental programs, several factors may explain the preferential selection for feedback interactions. First, autoregulatory and mutual feedback loops enable bistability, which helps maintain stable cell identity. Second, while feedforward loop cascades introduce response delays, they also amplify noise, whereas positive feedback circuits delay responses while maintaining noise robustness. This distinction suggests that feedback interactions are evolutionarily favored to enhance developmental stability and precision (51, 52).

### Limitations of the Study.

This study focuses on the design principles of developmental programs at the mRNA level, considering gene interactions at the transcription level. However, it does not address protein-level dynamics of morphogen signaling, which are also important for understanding developmental processes. Additionally, our framework provides theoretical predictions on the mesoscale structure of developmental GRNs from a systems-level perspective. Future experimental validation of key TFs and target gene roles within these networks will be important to complement and extend our findings.

## Methods

### Inference of GRNs from Single-Cell Data.

We analyzed scRNAseq data from Fawkner-Corbett et al. (46) using the Python implementation of SCENIC (pySCENIC) (47, 53). The pySCENIC pipeline was applied separately to the expression data from each developmental time point and consists of: 1) obtaining expression data for each time point using the Seurat pipeline (54); 2) inferring an adjacency matrix describing candidate coexpression relationships between TFs and target genes; and 3) constructing regulons, defined as modules containing a TF and its predicted direct target genes, by integrating cis-regulatory motif information. pySCENIC then uses AUCell to quantify regulon activity in each individual cell by calculating an AUC-based ranking score: genes are ranked by expression within each cell, and the score reflects whether the predicted target genes of a regulon are enriched among the highly expressed genes in that cell. Thus, regulon activity represents the relative activation of a TF-associated target-gene program, rather than the expression level of the TF alone. We repeated the pySCENIC pipeline six times and retained overlapping regulons to reduce noise associated with stochastic variation in the network-inference step. To focus on TF programs with variable activity in the tissue, we filtered out low-variance regulons by removing regulons with activity variance <0.1, where activity variance is defined as the variance of the AUCell regulon-activity score across cells. To build the GRNs, we considered TF-target pairs with predicted regulatory scores higher than the median across all pairs, considering genes that belong to a manually curated list of developmental-related genes (5,143 genes).

### Network Motif Enrichment Analysis.

To identify network motifs, statistically enriched patterns in the developmental regulatory networks, we used the MFinder package (55) to identify network motifs of up to 3 nodes. For each developmental GRN, motif enrichment was assessed relative to randomized networks that preserve the incoming and outgoing degree distributions of the original network. For each motif topology, we calculated a Z-score by comparing the number of motif occurrences in the real network to the distribution of motif counts in the randomized networks. Positive Z-scores indicate motifs that are enriched relative to the randomized networks, whereas negative Z-scores indicate underrepresented motif topologies, or anti-motifs. We verified these network motifs by detecting enriched patterns using the IGraphM package in Mathematica. To randomize the networks, we used the IGRewire function, and to find motifs and their frequencies we used the IGMotifs function. Because self-regulatory edges are constrained by the degree-preserving randomization procedure, autoregulation was quantified separately as the fraction of nodes with self-loops rather than by a Z-score. For all other motifs, enrichment and depletion were quantified using Z-scores relative to the randomized networks.

### Motif Role Transitions.

To explore transitions of genes between network motif roles, we first categorized the genes in the regulatory networks according to their role (or roles) in the motifs. We denote $N_{m,t}$ as the set of nodes in a GRN that participate in network motifs in time t. Next, for each time point t, we categorized nodes in $N_{m,t}$ to 7 groups according to their roles in the network motifs: $\left\{n_{1,t},n_{2,t},\ldots ,n_{7,t}\right\}$, for the 7 unique motif roles in our case (Fig. 3*A*). Note that certain nodes may belong to more than one group (which is the case where network motifs are combined). Next, for each one of the 7 motif roles, we consider all successive pairs of node groups within each role, such as $\left\{n_{1,t},n_{1,t+1}\},\;\{n_{1,t+1},n_{1,t+2}\right\}$, etc. For each pair of successive groups, we compute the Jaccard index: $J(n_{i,t},n_{i,t+1})=|n_{i,t}\cap n_{i,t+1}|/|n_{i,t}\cup n_{i,t+1}|$, which is the size of the intersection of the two groups divided by the size of their union. If all genes in a certain motif role remain in the same motif role in the next time point: J=1, and if none of the genes remain in the same motif role: J=0.

To build the network of motif role transitions, we defined motif role states for every gene (g) in the networks in each time point (t): $x_{g,t}$, which is a 7-dimensional vector that represents whether gene g participates in each one of the 7 motif roles or not (for example: (1,0,0,0,0,0,0),(1,1,0,0,0,0,0),(1,0,1,0,0,0,0), etc.). Overall, there are $2^{7}=128$ different possible motif role states (although not all of them are implemented in the developmental GRNs). We then build a network where the nodes represent the different possible motif role states. We then consider for every gene, all pairs of motif role states from successive time points:${\{x}_{g,t}\to x_{g,t+1}\}$. For each particular direct transition between motif role states, we draw an edge connecting the two motif role states (or a self-loop arrow in case the genes retain the same motif role state). We assign weights to the edges to account for the observed frequencies of each transition.

### Correlations between Network Motif Identities and Gene Expression Profiles.

We consider pairs of specific cell types (clusters) within the epithelial cells (and separately also within the fibroblast populations) across successive time points in the data. For each such pair of cell types and for every pair of successive time points, we compute the Jaccard index of genes participating in a certain network motif or are regulated by a certain network motif. For example, considering the feedforward loop (FFL), first we create a list of all the triplets of genes participating in FFLs in each cell type. We then compute the Jaccard index of the two lists of triplet genes. We compare the Jaccard index with the euclidean distance between the gene expression profiles of the two cell types in question. We then used the PearsonCorrelationTest function in Mathematica to estimate the Pearson correlation for every network motif and its statistical significance.

### Detection of Over- and Underrepresented Network Hypermotifs.

To detect over- and underrepresented network hypermotifs, we detect enrichment of particular combinations of network motifs. To do so, we follow the following steps (presented in detail in ref. 44):

1. Compute the Jaccard index for all k(k-1)/2 pairs of motif roles (here k=7) within each time point: $J\left(n_{i,t},n_{j,t}\right)=|n_{i,t}\cap n_{j,t}\left|/\right|n_{i,t}\cup n_{j,t}|.$
2. For nodes that appear more than once in the same role within the same network, we compute the Jaccard index by the ratio of the number of nodes that appear in a network motif role more than once to the total number of nodes that participate in that motif role.
3. We use the MFinder package (55) to create 100 random networks (for each time point in the data) that have the same number of nodes and edges, the same number of incoming and outgoing edges per node, and the same frequency of all subgraphs up to three-node subgraphs, as in the real network.
4. After categorizing the nodes according to their motif roles for each of the 100 random networks (per time point), we repeat steps 1 through 2 for all the random networks and compute $J_{\mathrm{rand}}\left(n_{i,t},n_{j,t}\right)$ for all pairs of motif roles in the random networks.
5. For every *i*,*j* such that i,j∈{1,2,…,k}, we compute the Z-score of $J(n_{i,t},n_{j,t})$ from the distribution of ${\{J}_{\mathrm{rand}}\left(n_{i,t},n_{j,t}\right)\}:Z_{\mathrm{ij}}=$$\left(J\left(n_{i,t},n_{j,t}\right)-mean{(\{J}_{\mathrm{rand}}\left(n_{i,t},n_{j,t}\right)\right\}))/std\left(\left\{J_{\mathrm{rand}}\left(n_{i,t},n_{j,t}\right)\right\}\right)$, and compute the *P* value by estimating the cumulative density of $Z_{\mathrm{ij}}$ for a normal distribution with zero mean and unit variance. We use the function NormalPValue in the package HypothesisTesting in Mathematica to compute the *P* value. We then use the Benjamini-Hochberg procedure to correct for multiple hypothesis testing, which provides us a corrected q-value for each pair of motif roles.
6. We consider over- and underrepresented motif combinations if their q-value is smaller than 0.05, where $Z_{\mathrm{ij}}>0$ for overrepresented combinations and $Z_{\mathrm{ij}}<0$ for underrepresented combinations.

### Mathematical Models for Statistically Enriched Network Hypermotif Regulatory Circuits.

We consider the following differential equations to model the dynamical behavior of the hypermotif circuit that combines two FFLs and a mutual feedback loop (Fig. 5*B*):

$$\frac{\mathrm{dx}}{\mathrm{dt}}=1-x,$$

$$\frac{\mathrm{dy}}{\mathrm{dt}}=\frac{y^{n_{\mathrm{yy}}}}{y^{n_{\mathrm{yy}}}+k_{\mathrm{yy}}^{n_{\mathrm{yy}}}}\frac{x^{n_{\mathrm{xy}}}}{x^{n_{\mathrm{xy}}}+k_{\mathrm{xy}}^{n_{\mathrm{xy}}}}\frac{w^{n_{\mathrm{wy}}}}{w^{n_{\mathrm{wy}}}+k_{\mathrm{wy}}^{n_{\mathrm{wy}}}}-y,$$

$$\frac{\mathrm{dw}}{\mathrm{dt}}=\frac{w^{n_{\mathrm{ww}}}}{w^{n_{\mathrm{ww}}}+k_{\mathrm{ww}}^{n_{\mathrm{ww}}}}\frac{x^{n_{\mathrm{xw}}}}{x^{n_{\mathrm{xw}}}+k_{\mathrm{xw}}^{n_{\mathrm{xw}}}}\frac{y^{n_{\mathrm{yw}}}}{y^{n_{\mathrm{yw}}}+k_{\mathrm{yw}}^{n_{\mathrm{yw}}}}-w,$$

$$\frac{\mathrm{dz}}{\mathrm{dt}}=\frac{x^{n_{\mathrm{xz}}}}{x^{n_{\mathrm{xz}}}+k_{\mathrm{xz}}^{n_{\mathrm{xz}}}}\frac{y^{n_{\mathrm{yz}}}}{y^{n_{\mathrm{yz}}}+k_{\mathrm{yz}}^{n_{\mathrm{yz}}}}\frac{w^{n_{\mathrm{wz}}}}{w^{n_{\mathrm{wz}}}+k_{\mathrm{wz}}^{n_{\mathrm{wz}}}}-z.$$

The model is formulated using dimensionless variables and parameters and is not fitted to kinetic measurements. Instead, parameter values are chosen to illustrate qualitative behaviors that can be generated by the inferred circuit topology. We chose parameter values that provide bistability to the mutual feedback circuit. This choice allows us to examine how the state of the feedback module affects the response of the downstream output gene Z. We therefore used the following parameter values:

$$k_{\mathrm{ww}}=k_{\mathrm{wy}}=k_{\mathrm{yw}}=k_{\mathrm{yy}}=0.3,\;k_{\mathrm{wz}}=k_{\mathrm{xw}}=k_{\mathrm{xy}}=k_{\mathrm{xz}}=k_{\mathrm{yz}}=0.01,$$

$$n_{\mathrm{ww}}=n_{\mathrm{wy}}=n_{\mathrm{yw}}=n_{\mathrm{yy}}=3,\;n_{\mathrm{wz}}=n_{\mathrm{xw}}=n_{\mathrm{xy}}=n_{\mathrm{xz}}=n_{\mathrm{yz}}=1.$$

We used the following initial conditions for Fig. 5*D*:

$y_{0}=0.25,w_{0}=0.35,z_{0}=0.01$, and $x_{0}$ as denoted in Fig. 5*D*.

and the following initial conditions for Fig. 5*E*:

$x_{0}=0,w_{0}=0.3,z_{0}=0.01$, and $y_{0}$ as denoted in Fig. 5*E*. Because these parameters are not experimentally constrained, the simulations should be interpreted as qualitative examples of behaviors enabled by the topology, rather than quantitative predictions of a specific in vivo circuit.

We consider the following differential equations to model the dynamical behavior of the hypermotif circuit that combines two FFLs forming a multi-input FFL (Fig. 5*F*):

$$\frac{\mathrm{dy}}{\mathrm{dt}}=\frac{x_{1}}{x_{2}}-y,$$

$$\frac{\mathrm{dz}}{\mathrm{dt}}=\frac{x_{1}x_{2}}{y}-z.$$

Here we used log linear terms (assuming that the variables are far from saturation) since this is the range of parameters that provide well-known dynamical properties of the coherent and incoherent FFLs such as the fold-change detection property (41, 56–59). This reduced model is used to illustrate how combining coherent and incoherent FFLs can generate differential sensitivity to two inputs. Other parameter regimes may support additional dynamic behaviors, and systematic experimental constraint of these parameters would require time-resolved perturbation measurements of the relevant TFs and target genes.

## Supplementary Material

Appendix 01 (PDF)

## Data Availability

Previously published data were used for this work (46).
