# Supplementary material for: Recurrent hypermotif regulatory circuits in developmental programs
Source: Proc Natl Acad Sci U S A. 2026 Oct 5;123(41):e2538056123. doi: 10.1073/pnas.2538056123 (PMC13663756; doi:10.1073/pnas.2538056123)
Supplement: Supplementary file 1 [file pnas.2538056123.sapp.pdf]

SI Text and Figures

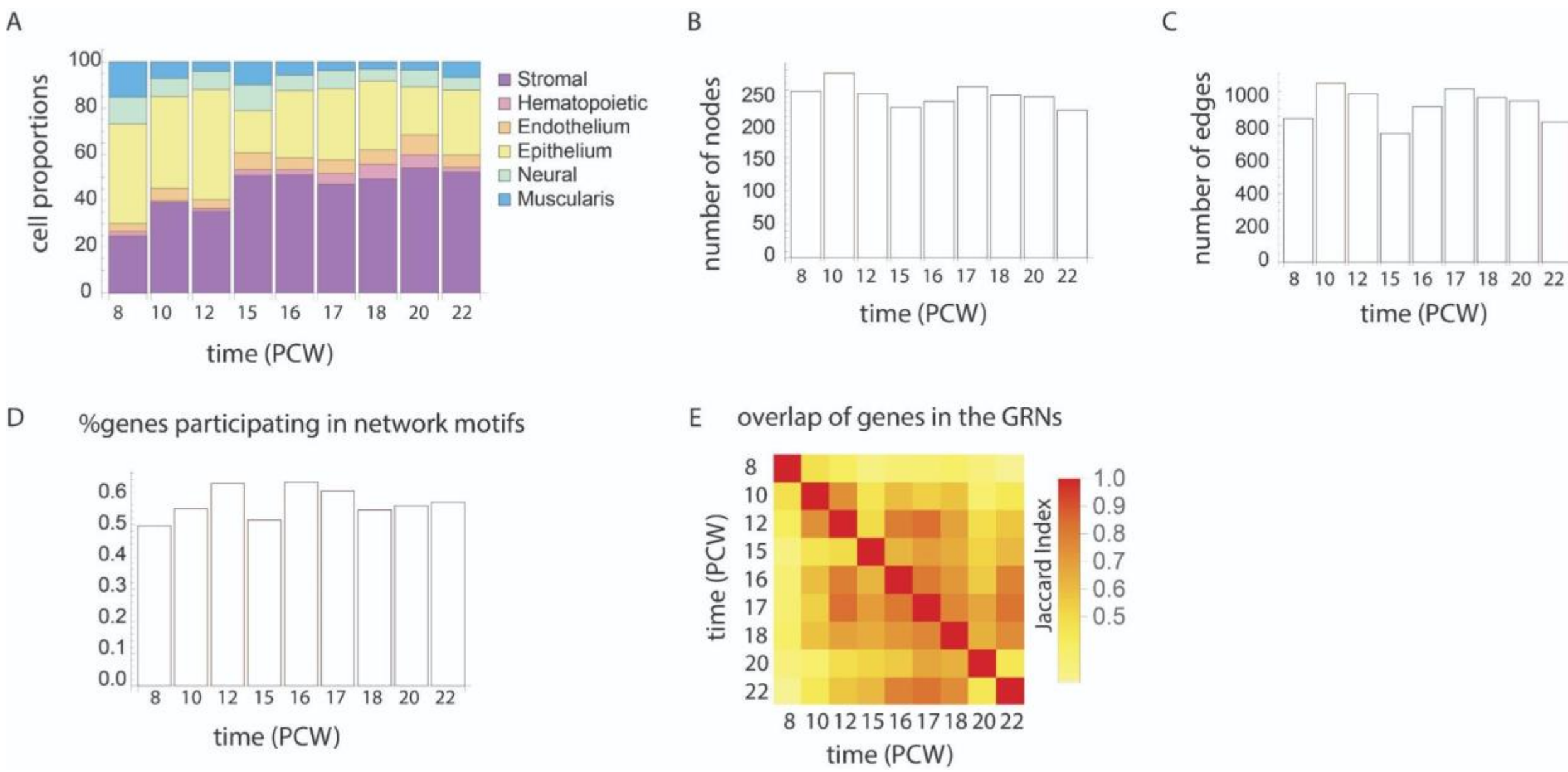

Figure S1: (A) Proportions of the 6 major cell type categories in the intestine computed from the single-cell data in Fawkner-Corbett et al.[46] across the 9 time points that follow the developmental process. (B-C) Number of nodes (B) and edges (C) in each network inferred from the single-cell data. (D) Proportion of genes that participate in network motifs out of the total number of genes in each network. (E) Heatmap of the overlap of genes that participate in the different gene regulatory networks (GRNs). Every square is a comparison between two networks from time points in the data. Overlap is computed by calculating the Jaccard index of the two lists of genes participating in the two networks.

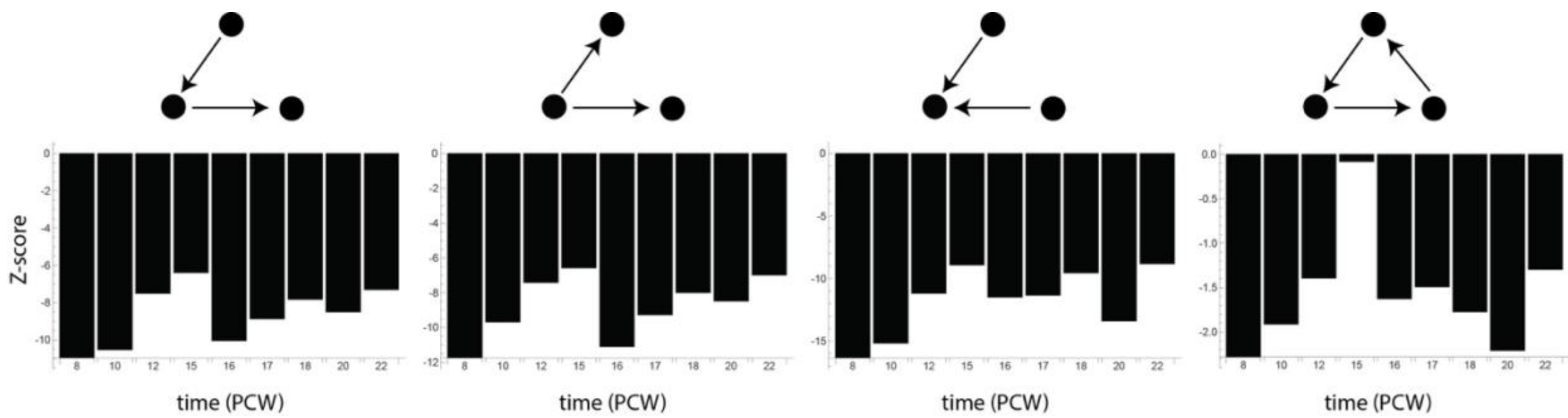

Figure S2: Underrepresented three-node motifs in developmental GRNs. Z-scores of four three-node motif topologies that are underrepresented across developmental time points relative to degree-preserving randomized networks. Negative Z-scores indicate motif depletion compared with randomized networks with the same incoming and outgoing degree distributions.

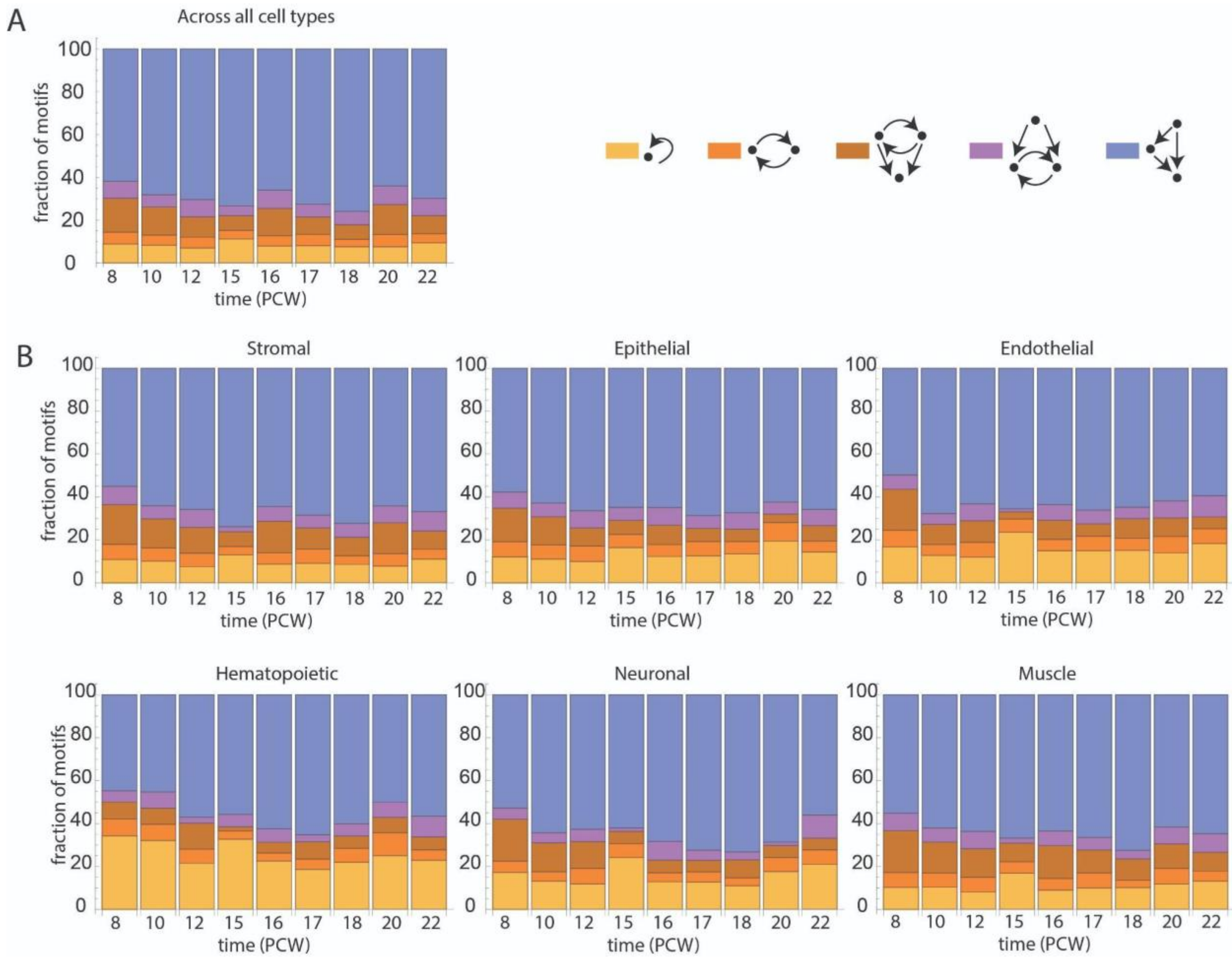

Figure S3: (A) Fraction of network motif regulatory circuits where we sum up interactions across all cell types. (B) Fractions of network motifs considering gene regulatory networks of each major cell type category separately.

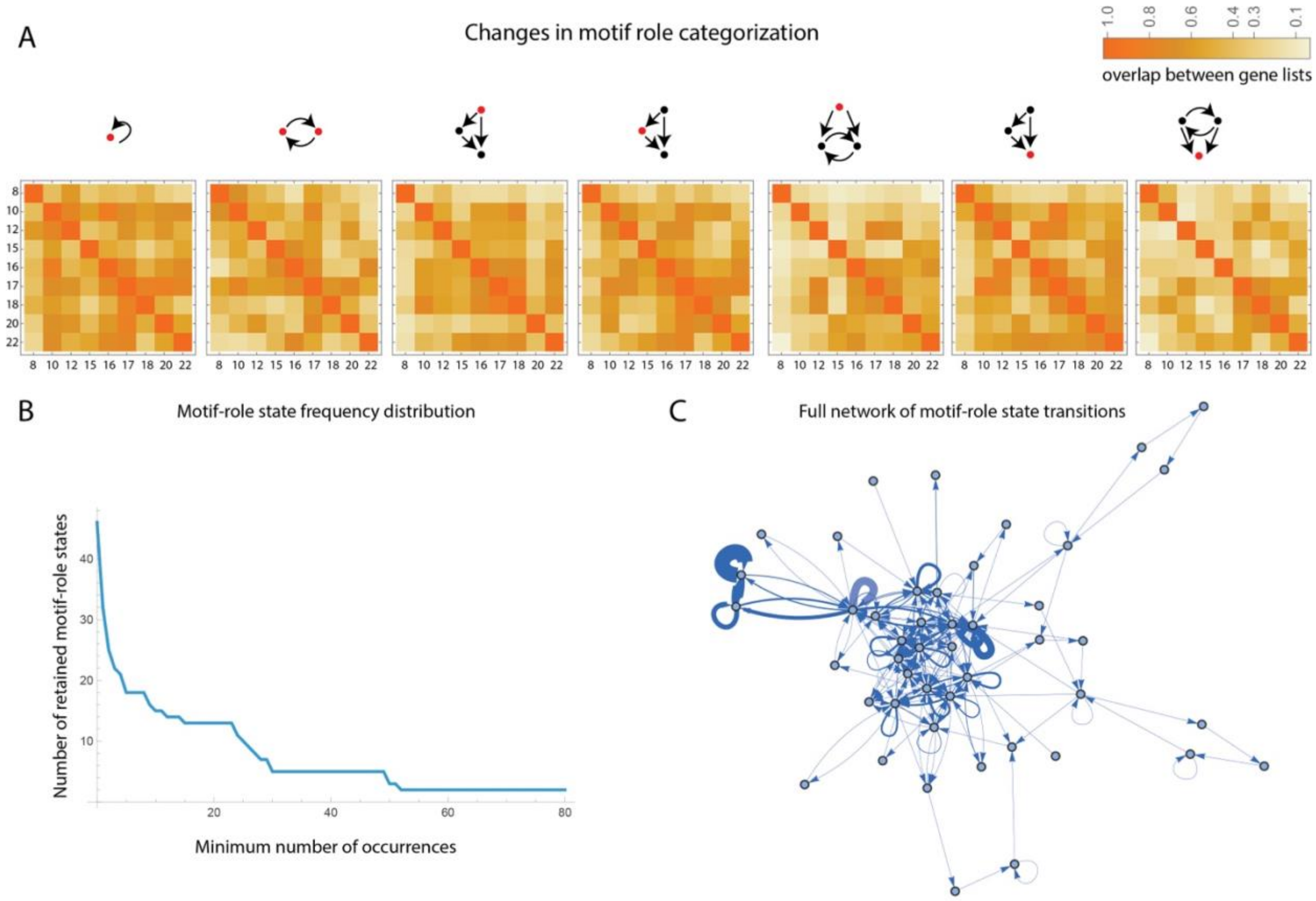

Figure S4: (A) Heatmaps showing the overlap between gene lists assigned to each motif role across all pairs of developmental time points, measured using the Jaccard index. Each panel corresponds to one motif role (marked by the red node in the circuit topology above each heatmap). (B) Number of unique motif-role states retained as a function of the minimum frequency threshold. Motif-role states are defined as binary combinations of the seven motif roles shown in Fig. 3A. Although $2^7 = 128$ states are theoretically possible, only 46 are observed at least once, and the number of recurrent states remains on the order of a few tens across a broad range of frequency thresholds. (C) Full unfiltered network of motif-role-state transitions across successive developmental time points. Nodes represent motif-role states and directed edges represent transitions between consecutive time points. Edge thickness reflects transition frequency. The unfiltered network contains many low-frequency transitions; therefore, the main Fig. 3G shows a filtered network including only transitions observed more than twice, highlighting recurrent transition patterns.
